# Motion Blur Kernel Rendering Using an Inertial Sensor: Interpreting the Mechanism of a Thermal Detector

**DOI:** 10.3390/s22051893

**Published:** 2022-02-28

**Authors:** Kangil Lee, Yuseok Ban, Changick Kim

**Affiliations:** 1 Agency for Defense Development, Daejeon 34060, Korea; traveler0802@kaist.ac.kr; 2 School of Electrical Engineering, Korea Advanced Institute of Science and Technology (KAIST), 291 Daehak-ro, Yuseong-gu, Daejeon 34141, Korea; 3 School of Electronics Engineering, Chungbuk National University, 1 Chungdae-ro, Seowon-gu, Cheongju 28644, Korea; ban@cbnu.ac.kr

**Keywords:** motion blur model, synthetic blurry thermal image, thermal detector, thermal image deblurring, blur kernel rendering, inertial sensor, gyroscope sensor

## Abstract

Various types of motion blur are frequently observed in the images captured by sensors based on thermal and photon detectors. The difference in mechanisms between thermal and photon detectors directly results in different patterns of motion blur. Motivated by this observation, we propose a novel method to synthesize blurry images from sharp images by analyzing the mechanisms of the thermal detector. Further, we propose a novel blur kernel rendering method, which combines our proposed motion blur model with the inertial sensor in the thermal image domain. The accuracy of the blur kernel rendering method is evaluated by the task of thermal image deblurring. We construct a synthetic blurry image dataset based on acquired thermal images using an infrared camera for evaluation. This dataset is the first blurry thermal image dataset with ground-truth images in the thermal image domain. Qualitative and quantitative experiments are extensively carried out on our dataset, which show that our proposed method outperforms state-of-the-art methods.

## 1. Introduction

Infrared images are increasingly being used in various fields, e.g., commercial, medical, and military applications. Infrared cameras have been mainly used in industrial applications, such as thermal insulation performance measurement and electrical leakage testing [1]. Recently, new applications of infrared imaging are emerging. For instance, drones equipped with infrared cameras have been used to search for missing survivors at nighttime [2,3], and the infrared camera is becoming an essential sensor for autonomous vehicle driving at night to prevent accidents [4]. Furthermore, due to the outbreak of COVID-19, many applications measuring the body temperature of visitors at a building entrance have been widely used.

The infrared image sensor is a device that displays the thermal information of subjects as an image. The wavelength of the infrared band is longer than the visible band, being invisible to human eyes. The infrared band can be categorized into three types according to its wavelength: Short Wavelength Infrared (SWIR) with the wavelength ranging from 1.4 μm to 3 μm, Mid Wavelength Infrared (MWIR) with the wavelength ranging from 3 μm to 8 μm, and Long Wavelength Infrared (LWIR) with the wavelength ranging from 8 μm to 15 μm [5]. Due to the cost issue, most commercial applications use LWIR image sensors. More specifically, since SWIR and MWIR image sensors are fabricated based on compound semiconductors, they are more expensive than silicon-based visible and LWIR image sensors. Further, MWIR image sensors require a cryogenic system to maintain the sensor temperature at precisely 77K, which significantly increases the price, volume, and weight. Therefore, the MWIR image sensors have limitations in being used for commercial purposes. The cost of LWIR image sensors, on the other hand, is relatively low because they are fabricated based on the MEMS (Micro Electro Mechanical Systems) technology. Further, the LWIR image sensors can be manufactured in a very small since they do not need any cryogenic cooling system. The principle of the LWIR image sensors are different from the ones of CCD and CMOS image sensors which usually are for visible band images. The CCD and CMOS image sensors, so-called photon detectors, have semiconductor materials and structures that directly convert photons into electrons. In contrast, the LWIR sensors have the structure of a microbolometer [6]. This structure absorbs photons and changes them into heat. The LWIR sensors generate an image signal by detecting the temperature change induced by photons. The sensors having the mechanism of a microbolometer are called thermal detectors.

Traditional image processing tasks such as denoising [7,8,9,10], contrast enhancement [11], deblocking [12,13], inpainting [14,15], deblurring [16,17,18,19], and compressive sensing recovery [20,21] have been intensively studied in the visible image area since it is easy to acquire sufficient test data. However, due to domain dependency, image processing algorithms that properly work on a visible image are not guaranteed to work well on a thermal image. In general, the algorithms developed for the visible images tend to suffer from performance degradation in the thermal image domain. Therefore, it is essential to develop algorithms that directly consider the characteristics of the image domain. For example, in the studies on image quality metric, many efforts have been made to find appropriate metrics for thermal images [22,23,24]. Further, in the studies on image enhancement, many research proposals have been made to develop methods specialized for thermal images to solve problems such as low signal-to-noise ratio (SNR), halo effect, blurring, and low dynamic range compared to visible images [25,26,27].

**Figure 1:**
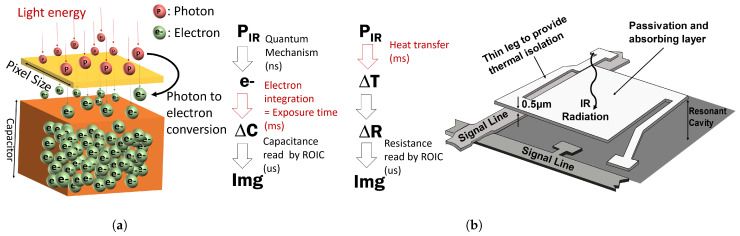
The mechanism of two different sensors and cause of motion blur. (**a**) the cause of motion blur in the photon detector is integration time, (**b**) the cause of motion blur in the thermal detector is the response time of temperature change.

**Figure 2:**
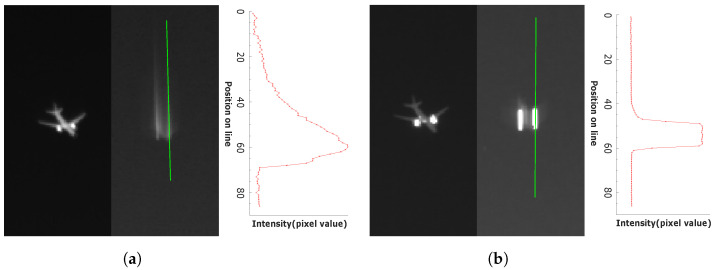
Two kinds of cameras simultaneously take an image of the aircraft’s twin-jet engine flames. Both images have motion blur, but they have different motion blur patterns. (**a**) LWIR camera using thermal detector, (**b**) MWIR camera using photon detector.

The domain dependency can also be observed in the image deblurring area, where the two types of sensors produce apparently different motion blur patterns. The shape of a motion blur is very strongly related to the principle of image sensors, as shown in Figure 1. Photon detectors such as CCD and CMOS require time to physically collect photons, which is called exposure time (or integration time). If the camera or subject moves during the exposure time, motion blur occurs in the resulting image. In addition, the motion blur is easily observed at nighttime when the camera needs a longer exposure time. In contrast, the main cause of the motion blur in thermal detectors is the heat flow in a microbolometer structure. The microbolometer structure is designed and manufactured to provide good thermal isolation. Due to the thermal isolation of the microbolometer, time is needed for the heat to be transferred from one structure to another. The thermal detector generates images by measuring the temperature change of a microbolometer structure. Therefore, the remaining heat in the previous frame can appear as the motion blur in the next frame. As such, the photon detector and the thermal detector have different mechanisms for motion blur and produce different blur patterns in an image. As shown in Figure 2, the motion blur of the photon detector exhibits a linear blur pattern, whereas the thermal detector shows a blur pattern similar to a comet-tail shape.

Several algorithms have been proposed to address this issue for thermal image deblurring. Oswald-Tranta [28] and Nihei et al. [29] observed that the motion blur in the LWIR image is different from that of the visible image and then proposed methods for image restoration. However, their image restoration experiments were conducted in limited conditions. The target’s velocity was maintained with a constant at a fixed distance from the sensor, or the camera moved at a constant speed with its fixed direction. Consequently, their deblurring methods suffer from performance degradation when the size or orientation of the motion blur changes. Ramanagopal et al. [30] assumed the temporal sparsity of pixel-wise signals and performed motion deblurring on a thermal video using the LASSO (Least Absolute Shrinkage and Selection Operator) algorithm. However, it does not operate in real-time, and the deblurring fails when the temporal sparsity assumption is broken (e.g., fast camera motion). Zhao et al. [31] used the deep learning-based approach, a new GAN (Generative Adversarial Networks) structure for thermal image deblurring. However, the training dataset was synthesized simply by averaging video frames without considering the characteristics of a motion blur in thermal images. Therefore, their method cannot be applied to thermal images with large motion blur. Batchuluun et al. [32] improved the deblurring performance by converting the one-channel thermal image into a three-channel thermal image. However, their method also did not consider how the motion blur occurs in thermal images when constructing the training dataset.

In fact, a number of deblurring methods have been studied based on visible images. Deep-learning-based methods have recently shown state-of-the-art performance in the image deblurring task, outperforming classic handcrafted methods. LSTM and CNNs are combined in SRN-DeblurNet [33] to deblur an image in a multi-scale manner. Pan et al. [34] proposed a method, in which neighboring video frames are warped into the center frame to use latent image information from adjacent frames for deblurring. Kupyn et al. [35] proposed a GAN-based structure, in which the feature pyramid networks balance performance and efficiency. Ye et al. [36] proposed a scale-iterative upscaling network with sharing weights to recover sharp images, and they used the super-resolution architecture for better performance. Zha et al. [18] proposed an effective algorithm for image deblurring by combining an optimization-based model with a deep neural network model. Although the deep learning-based method shows remarkable performance, the deblurring performance can still be significantly improved by incorporating the thermal image characteristics as well as by addressing the issue of the lack of datasets. Except for deep learning-based approaches, the most common and widely used approach for image deblurring is to estimate the blur kernel and sharp image simply using the observed blurry image [16,17,19]. In these conventional methods, the latent image and blur kernels are obtained by minimizing the energy function with its constraints of statistics information. However, as a typical ill-posed problem, the conventional methods need large computational resources and often fail to deblur when the blur kernel size is large. So as to avoid these problems, the approach using an inertial sensor has been proposed especially for the blurry images caused by camera motions [37,38,39,40,41,42,43,44,45,46,47]. This approach has been evaluated as a method with great advantages over the existing blind deblurring method, in that the computational resources can be reduced by directly rendering the blur kernel with the inertial sensor information. However, all previous studies have proposed blur kernel rendering methods based on a photon detector model, which is generally used for visible images.

This paper proposes a novel motion blur kernel rendering method inspired by the sensing mechanism of a thermal image sensor and the supplementary information from a gyroscope sensor. Rendering the blur kernel by using gyroscope information is both efficient and accurate. It also enables the deblurring task through an efficient deconvolution. In our study, we interpret the microbolometer structure model in the aspect of motion blur, construct the motion blur model of the thermal image, and propose the method to efficiently and accurately render a blur kernel connoting the properties of the physical mechanism.

The main contributions of our study are summarized as follows:

- We propose a novel synthesis method for the blurring effect in the thermal image by interpreting the operating properties of a microbolometer.
- We propose the blur kernel rendering method for a thermal image by combining the gyroscope sensor information with the motion blur model.
- We acquire and publically release both actual thermal images and synthetic blurry thermal images for the construction of a dataset for thermal image deblurring.
- Our method quantitatively and qualitatively outperforms the latest state-of-the-art deblurring methods.

## 2. Image Generation and Motion Blur Model

There is a fundamental difference between a photon detector and a thermal detector in the principle of image generation. This section describes the mechanism of how the two detectors generate an image. Based on the analysis of detector mechanism, we propose an approach to synthesize the motion blur in a thermal image.

### 2.1. Photon Detector Model

A photon detector is based on a photodiode structure. When photons are incident on the p–n junction in the photodiode, electron-hole pairs are generated, and the electrical current flows along with the direction of the photodiode bias. The generated electrons are accumulated in a capacitor during the integration time. The integration time means the exposure time of a camera. The read-out integrated circuit (ROIC) outputs an image signal by measuring the charge stored in the capacitor.

(1) $$I\left(i,j\right)=\int_{0}^{T_{int}}\Phi_{i,j}\left(t\right)dt.$$

As can be seen in Equation (1), an image is corresponds to the sum of the incident photon energy during the integration time. The incident photon power is $\Phi_{i,j}\left(t\right)$, the image signal is I(i,j), and the integration time is $T_{int}$, where (i,j) is the index of pixels in an image. Previous studies have used Equation (2) to generate a motion blur image from sharp images in the visible image domain [48,49,50,51].

(2) $$B\left[n\right]=\frac{1}{n}\sum_{k=1}^{n}S\left[k\right].$$

S[k] denote the *k*th sharp image, which is equal to the incident photon power. *n* is the number of sampled sharp images during the exposure time.

### 2.2. Thermal Detector Model

**Figure 3:**
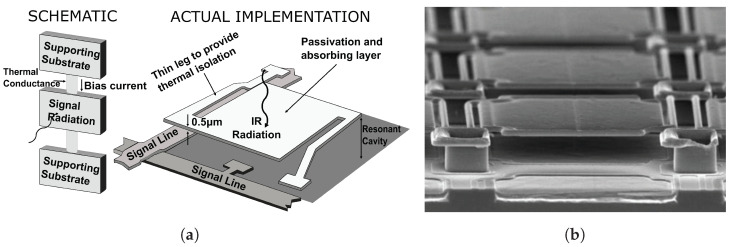
(**a**) Microbolometer structure and Schematic model, (**b**) Microbolometer scanning electron microscope (SEM) image [53].

The microbolometer sensor is the most frequently used device structure in a thermal detector. Since the fabrication cost of the microbolometer is relatively cheap than other structures, this structure is predominantly used for the mass-production of the uncooled infrared detector [6]. The operating mechanism of a microbolometer consists of four steps: (i) the incident photon energy is converted into thermal energy, (ii) the heat changes the device resistance, (iii) ROIC measures the amount of change in resistance, (iv) ROIC outputs an image signal proportional to the measuring value. The thermal isolation structure is essential for this four-stage operation to be conducted normally. The microbolometer supports a large sheet area with extremely thin legs for thermal isolation. The large sheet absorbs incident photons, and the generated heat is isolated by thin legs. The conceptual diagram of a microbolometer structure and substantive implementation are shown in Figure 3. The following Equation (3) expresses the heat flow of a microbolometer [52].

(3) $$C_{th}\;\cdot \;\frac{d\Delta T}{dt}+\frac{d\Delta T}{R_{th}}=\eta \Phi \left(t\right).$$

$C_{th}$, $R_{th}$, Φ(t), ΔT and η denote thermal capacitance (W·K), thermal resistance (K·W${}^{-1})$, photon power (*W*), device temperature (*K*) and photon absorption rate, respectively. $C_{th}R_{th}$ is the thermal time constant value and is expressed as τ. Therefore, Equation (3) becomes Equation (4), and the solution of first-order differential equation is given as Equation (5).

(4) $$\tau \;\cdot \;\frac{d\Delta T}{dt}+\Delta T=R_{th}\eta \Phi \left(t\right),$$

(5) $$\Delta T\left(t\right)=\frac{R_{th}\eta }{\tau }\Phi \left(t\right)*e^{\frac{-t}{\tau }}.$$

Let B(t) be a final output image. The temperature difference is converted into an image signal through the element resistance change. As a more specific expression, the temperature difference of the microbolometer and the signal level of an output image are proportional to each other [6]. Therefore, considering the scale factor, Equation (5) is expressed as Equation (6).

(6) $$\begin{array}{c} B\left(t\right)=K\Phi \left(t\right)*e^{\frac{-t}{\tau }},\;\mathrm{where}\;K={\frac{R_{th}\eta }{\tau }}_{.} \end{array}$$

It is important to note that the image generation models of a thermal detector and a photon detector are different as shown in Equations (6) and (1). In the case of the photon detector, the output signal is formed by accumulating incident photon energy. On the other hand, the output of the thermal detector is the convolutional result of incident photon energy and an exponential decay function. Therefore, the output images of the thermal detector lose the signal value over time. The theoretical mechanism difference between the two detectors is observed by our experiments. Even though the photon detector and thermal detector acquire a moving subject simultaneously, the blur effects appear differently, as shown in Figure 2. The response time of the thermal detector is related to τ. A high τ value means that the device has a high response time, showing a large amount of motion blur in an image. In contrast, a low τ value indicates less amount of blur effect in an image due to the faster response of the device.

### 2.3. Generating the Synthetic Blurry Image in a Thermal Image

In order to actually use the thermal detector model, it is necessary to convert the continuous model into a discrete model. Therefore, for the discrete model, we propose a new assumption based on Equation (4). A sampling process is used to replace continuous-time with discrete-time. Through the sampling process, *t* is converted to $t_{k}$. By applying Backward Euler method [54], Equations (7)–(9) can be obtained based on Equation (4) using $\frac{d\Delta T(t_{k})}{dt_{k}}\approx \frac{\Delta T\left(t_{k}\right)-\Delta T\left(t_{k-1}\right)}{h}$.

(7) $$\tau \;\cdot \;\frac{\Delta T\left(t_{k}\right)-\Delta T\left(t_{k-1}\right)}{h}+\Delta T\left(t_{k}\right)=R_{th}\eta \Phi \left(t_{k}\right),$$

(8) $$\begin{array}{cc} \Delta T(t_{k}) & =\frac{\tau }{\tau +h}\Delta T\left(t_{k-1}\right)+\frac{h}{\tau +h}\Phi^{{}^{\prime }}\left(t_{k}\right),\\ & \mathrm{where}\;\Phi^{{}^{\prime }}\left(t_{k}\right)=R_{th}\eta \Phi \left(t_{k}\right), \end{array}$$

(9) $$\begin{array}{cc} \Delta T(t_{k}) & =\left(1-\alpha \right)\Delta T\left(t_{k-1}\right)+\alpha \Phi^{{}^{\prime }}\left(t_{k}\right),\\ & \mathrm{where}\;\alpha ={\frac{h}{\tau +h}}_{.} \end{array}$$

$\Delta T(t_{k})$ is proportional to $B(t_{k})$, and $\Phi^{{}^{\prime }}\left(t_{k}\right)$ is a sharp image, which can be rewritten by using $S(t_{k})$. Furthermore, the formula for a single device can be expanded to an image array, and the formula should be as the following Equation (10).

(10) $$B_{i,j}\left(t_{k}\right)=\left(1-\alpha \right)B_{i,j}\left(t_{k-1}\right)+\alpha S_{i,j}\left(t_{k}\right).$$

The *k*th blurry image is expressed as the weighted sum of the blurry image at $t_{k-1}$ and the sharp image at $t_{k}$. Equation (10) has the form of the Infinite Impulse Response (IIR) filter, and when the recursive term is eliminated, it becomes Equation (11).

(11) $$B_{i,j}\left(t_{k}\right)=\alpha \sum_{n=1}^{k}{\left(1-\alpha \right)}^{k-n}S_{i,j}\left(t_{n}\right).$$

The blurry thermal image $B_{i,j}\left(t_{k}\right)$ is expressed as the exponential average of sharp images $S_{i,j}\left(t_{n}\right)$. In a photon detector, sharp images are averaged over a certain exposure time to synthesize a blurry image, as shown in Equation (2). On the other hand, it can be observed that an exponential average is used for a thermal image.

One thing that remains is how many sharp images are needed to synthesize the exact motion blur effect in the thermal detector. To address this problem, we need to look at the assumption taken in Equation (7). In the Backward Euler method, it is assumed that $h=t_{k}-t_{k-1}\approx 0$, while *h* is the interval time between $t_{k}$ and $t_{k-1}$. If the assumption $t_{k}\approx t_{k-1}$ is satisfied, then $\Phi \left(t_{k}\right)\approx \Phi \left(t_{k-1}\right)$ also must be satisfied. Therefore, to satisfy $\Phi \left(t_{k}\right)\approx \Phi \left(t_{k-1}\right)$, the translation using a sharp image must be less than one pixel during *h*. In other words, if the subject image focused on the sensor plane moves within one pixel during *h*, the subject does not change in the image. The assumption can be satisfied if the shift between adjacent images is within one pixel. For example, if the camera rotation directly causes an image motion blur, the following Equation (12) must be satisfied.

(12) $$h=t_{k}-t_{k-1}\leq {\frac{IFOV}{\omega }}_{.}$$

Instantaneous Field of View (IFOV) [55] is the field of view corresponding to a single pixel. ω is the angular velocity, which can be obtained when the camera rotates in the pitch or yaw direction. IFOV/ω is the time for an image to be shifted by one pixel. For example, if IFOV is 0.1° and the angular velocity of a camera is 100°/s, time interval *h* required for synthesis is 1 ms (where *h* is 1 ms, having the sharp image frame rate as 1000 Hz).

### 2.4. Verification of Thermal Detector Blur Model

This section describes the verification of our thermal detector blur model through experiments. Two test patterns are acquired using FLIR A655sc thermal camera and a collimator. Firstly, A655sc thermal camera was installed on the pan/tilt mount and rotated to collect real blurry images. Sharp images are obtained when the camera is stopped. The blurry images are synthesized by applying our thermal detector blur model to the sequential frames of sharp images. The model verification is achieved by quantitatively comparing real blurry images with synthetic blurry images.

#### 2.4.1. Acquiring a Real Blurry Image

**Figure 4:**
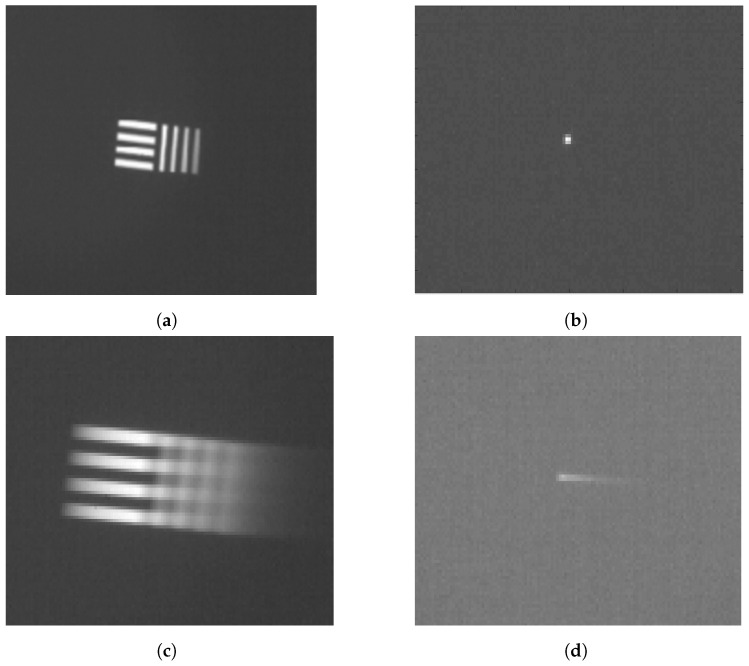
Examples of motionless and moving pattern images. (**a**) 4-bar pattern, (**b**) Point source, (**c**) 4-bar pattern at 40°/s, (**d**) Point source at 40°/s.

Real blurry images are acquired by rotating the camera at a certain angular velocity. The infrared camera is installed on a pan/tilt framework to precisely control the rotation speed. The image sensor plane is aligned with the rotation center. The camera rotation speed is 40°/s. Point source and 4-bar patterns are used as simple targets. The test patterns in a sharp image and a real blurry image are shown in Figure 4c,d, respectively.

#### 2.4.2. Obtaining a Synthetic Blurry Image from Sharp Images

**Figure 5:**
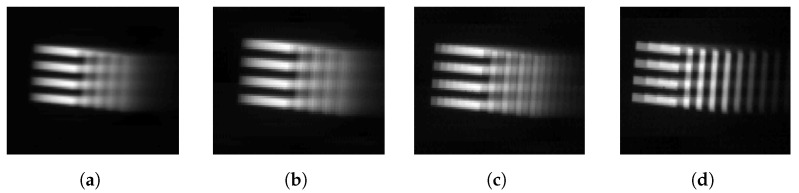
Examples of stepping effects. (**a**) Shifting one pixel between adjacent frames, (**b**) Shifting two pixels between adjacent frames, (**c**) Shifting four pixels between adjacent frames, (**d**) Shifting eight pixels between adjacent frames.

The set of sharp images with a high frame rate is required to generate synthetic blurry images via Equation (10). According to the previous section, a set of sharp images must be shifted by less than one pixel from adjacent frames. As shown in Figure 4a,b, we acquire a sharp image while the camera is stopped, and the set of sharp images is generated by shifting the image. The set of sharp images is used as $S_{i,j}\left(t_{k}\right)$ in Equation (10). If the sharp images are shifted by more than one pixel, the synthetic blurry image suffers from the stepping effect, as shown in Figure 5. The stepping effect makes synthetic blurry images have low similarity with real blurry images and makes them difficult to use either for training or for evaluation. In this experiment, the maximum rotation speed of a camera is 40°/s, and IFOV of FLIR A655sc is 0.0391°. Hence, the time interval *h* is 0.978 ms for synthesizing a blurry image without any stepping effect.

#### 2.4.3. Comparing Real and Synthetic Blurry Images

**Figure 6:**
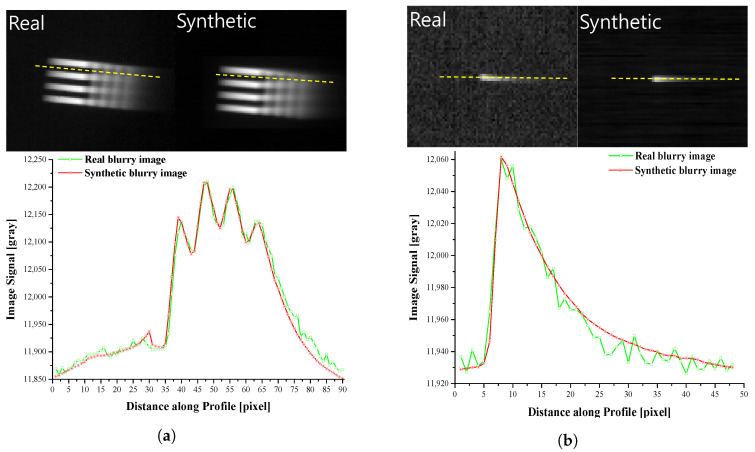
The comparison of real blurry images and synthetic blur images. (**a**) 4-bar pattern, (**b**) Point source.

Figure 6 shows the real and synthetic blurry images when the camera rotation speed is 40°/s. In both test patterns, the comet tail shape appears in the opposite direction of a target movement. Even though the camera is rotating at a constant speed, the asymmetric blur phenomenon occurs. There is no difference in the position and value of the peak point of a signal value between real and synthetic blurry images. Therefore, the two signal profiles show high similarity, which means that our model has the sufficient ability to synthesize a blur effect.

## 3. Blur Kernel Rendering Using a Gyroscope Sensor for a Thermal Detector

The gyroscope sensor provides reliable information for rendering the blur kernel in the blurry images caused by camera motions. The blur kernel rendering methods with the assistance of an external sensor have been studied in many papers [37,38,39,40,41,42,43,44,45,46,47]. However, all approaches have been conducted in the visible image domain based on a photon detector. We propose the first blur kernel rendering method using an inertial sensor in the thermal image domain, leveraging the physical model of a thermal detector.

### 3.1. Blur Kernel Rendering and Gyroscope Data Selection

When a camera has motion, the relationship between the real-world scene and the image on a camera sensor plane is expressed as a homography transform [56]. In this case, the camera motion is expressed by translation and rotation. The intrinsic matrix of a camera is expressed in Equation (13), where *f* is the focal length, ($p_{x_{0}}$,$p_{y_{0}}$) is the principal point, and *s* is the skew parameter.

(13) $$\left[\begin{array}{ccc} f & s & p_{x_{0}}\\ 0 & f & p_{y_{0}}\\ 0 & 0 & 1 \end{array}\right]$$

We assumed the principle point and skew parameter to be 0. If the distance between a camera and a target is *d*, the rotation matrix is R(θ), the translation vector is t, and the normal vector of a scene is n. Then, the warping matrix and the rotation matrix are expressed by Equations (14) and (15), respectively.

(14) $$H\left(t,\theta \right)=K\left(R\left(\theta \right)-\frac{tn^{⊺}}{d}\right)K^{-1},$$

(15) $$R\left(\theta \right)=\left[\begin{array}{ccc} cos\;\theta_{x} & -sin\;\theta_{x} & 0\\ sin\;\theta_{x} & cos\;\theta_{x} & 0\\ 0 & 0 & 1 \end{array}\right]\;\cdot \;\left[\begin{array}{ccc} cos\;\theta_{y} & 0 & sin\;\theta_{y}\\ 0 & 1 & 0\\ -sin\;\theta_{y} & 0 & cos\;\theta_{y} \end{array}\right]\;\cdot \;{\left[\begin{array}{ccc} 1 & 0 & 0\\ 0 & cos\;\theta_{z} & -sin\;\theta_{z}\\ 0 & sin\;\theta_{z} & cos\;\theta_{z} \end{array}\right]}_{.}$$

If the distance between a subject and a camera is longer than the focal length, the camera rotation is the dominant factor in the warping matrix rather than camera translation [57,58,59]. Therefore, according to the above assumption, Equation (14) can be approximated as Equation (16).

(16) $$H\left(\theta \right)=KR\left(\theta \right)K^{-1}.$$

It is reported in several studies that the path of a light point source, which is called a light streak in blurry images, corresponds to the shape of a blur kernel [60]. Generally, the blur kernel is expressed as the cumulative sum of unit impulse functions during the exposure time *T* in a camera using the photon detector. Therefore, the relationship between a camera motion and a blur kernel is as the following Equation (17). δ[x,y] is the unit impulse function, $f_{g}$ is the gyroscope frame rate, and $N_{p}$ is the total number of gyroscope data during the exposure time.

(17) $$\begin{array}{cc} k_{p}\left[x,y\right] & =\frac{1}{N_{p}}\sum_{i=1}^{N_{p}}\delta \left[x-x_{i},y-y_{i}\right],\\ \mathrm{where}\;(x_{i},y_{i},1) & =KR\left(\theta \left(t_{i}\right)\right)K^{-1}\left(x_{0},y_{0},1\right),\;N_{p}=Tf_{g}. \end{array}$$

**Figure 7:**
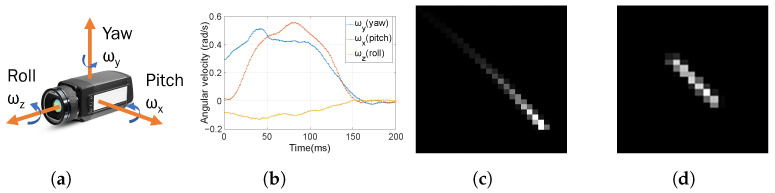
Illustration of camera rotation. (**a**) 3-axis rotation model, (**b**) Rotation motion measured by gyroscope sensor, (**c**) Blur kernel rendering result using the thermal detector model, (**d**) Blur kernel rendering result using the photon detector model.

The warping matrix of a thermal detector is identical to that of a photon detector case, but their image generation models are different. The blur kernel rendering method in the thermal image domain is expressed in Equation (18) by combining Equations (11) and (16). Since the exponential decay term causes the signal attenuation effect in Equation (18), the result of blur kernel rendering resembles a comet tail shape. Figure 7 shows the camera axis and the blur kernel rendering results. Since the position of a point source transformed through the warping matrix is not expressed as an integer, the bi-linear interpolation is conducted. ($1-{\left(1-\alpha \right)}^{N_{t}}$) is the normalization term to make the summation of the blur kernel be one. $f_{g}$ and $N_{t}$ are the gyroscope frame rate and the total number of gyroscope data during mτ in Equation (17), respectively.

(18) $$\begin{array}{cc} k_{t}\left[x,y\right] & =\frac{\alpha }{\left(1-{\left(1-\alpha \right)}^{N_{t}}\right)}\sum_{i=1}^{N_{t}}{\left(1-\alpha \right)}^{N_{t}-i}\delta \left[x-x_{i},y-y_{i}\right],\\ \mathrm{where}\;N_{t} & =m\tau f_{g}. \end{array}$$

The rotation matrix is required to implement the formula of blur kernel rendering. The angular information of each axis in the rotation matrix can be obtained through the gyroscope sensor. Since the gyroscope is a sensor that measures the angular velocity, the angle can be calculated by integrating the measured values over time. Next, we should decide the number of gyroscope data. In the case of a photon detector, the number of gyroscope data is easily determined by the exposure time, which induces the blur effect. In contrast, the blur effect of a thermal detector is caused by the thermal time constant in the microbolometer structure. Therefore, it is necessary to define the number of gyroscope data based on the thermal time constant τ. According to the modeling result in Equation (18), All gyroscope data stored during the entire duration are required for blur kernel rendering. However, the practical length of gyroscope data for rendering is limited due to the signal attenuation characteristics of the thermal detector. We confirmed that it is sufficient if the length of gyroscope data is at least five times the thermal time constant, or m=5. For instance, if τ is 8 ms, obtaining gyroscope data for 40 ms is enough to synthesize the blur kernel.

### 3.2. Calibration and Blur Kernel Refinement

**Figure 8:**
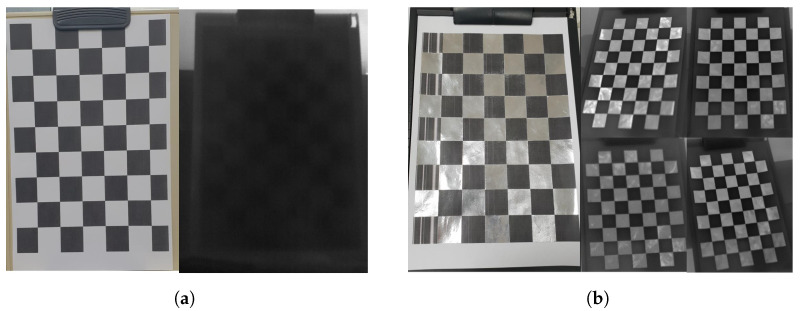
The calibration pattern for a thermal signal. (**a**) An ordinary checkerboard pattern (captured in visible-band and infrared band), (**b**) The checkerboard pattern improved by attaching aluminum material (captured in visible-band and infrared band).

We calibrate a camera and a gyroscope using the open-source code for calibration [61]. Generally, the calibration process can be conducted by a standard checkerboard pattern in a visible image. On the other hand, the thermal camera cannot display a standard checkerboard pattern without temperature variations. To solve this problem, we use aluminum tapes whose emissivity is different from that of paper, as shown in Figure 8.

**Figure 9:**
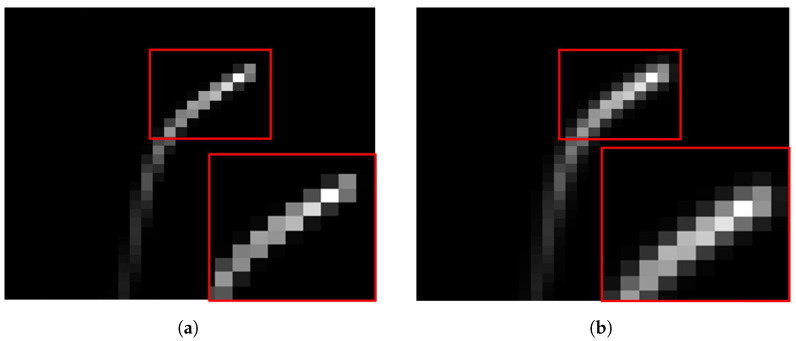
(**a**) Blur kernel before refinement, (**b**) blur kernel after refinement (given λ = 10 μm, f/♯ = 1.0, β = 0.6).

We conduct the refinement process for synthesizing the blur kernel as realistic as possible. The uniform blur effect appears even if there is no camera movement due to the optical Point Spread Function (PSF). The optical PSF is known to occur due to the diffraction and aberration of a camera lens system. Even for an ideal point source, a blur spot appears on the sensor plane by optical PSF [62]. Since diffraction increases as wavelength increases, the optical PSF is larger in an infrared band than in a visible band. Then, a refinement process considering the optical system is necessary to utilize the blur kernel rendering method in the infrared band. Precise optical measurement systems are required to synthesize an accurate optical PSF. However, these systems consume enormous time and cost. Instead, an efficient approximation formula is used in our method. As the primary cause of optical PSF, the diffraction blur spot size is expressed as an airy disk function. The airy disk equation is approximated as Gaussian function, and its standard deviation is expressed by Equation (19) [63].

(19) $$\sigma =0.45\;\cdot \;{\frac{\lambda \;\cdot \;f/♯}{\beta }}_{.}$$

where (19), λ is the infrared wavelength, f/♯ is the F-number, and β is the weighting factor to reflect the optical aberration effect. When β is 1, it directly means a diffraction-limited lens with no optical aberration effect. We determined the value of β with reference to the Strehl ratio to apply the optical aberration effect. Here, the Strehl ratio is defined as the peak intensity ratio of the center between a real PSF and an ideal PSF without aberrations [64]. Finally, the refined blur kernel can be calculated through the convolution between the blur kernel rendering result and the Gaussian function with the deviation value as σ shown in Equation (19). The blur kernel refinement results are presented in Figure 9.

## 4. Experimental Setup

### 4.1. Construction of Synthetic Blurry Thermal Image Dataset

Most of the datasets for evaluating deblurring performance consist of visible band images, while thermal image datasets with ground truth images cannot be found. In this paper, we introduce the first Synthetic Blurry Thermal Image (SBTI) dataset with ground truth images in the thermal image domain. Firstly, we constructed the Sharp Thermal Image (STI) dataset using FLIR A655sc LWIR camera. The gyroscope sensor was mounted on the camera to measure the camera rotation speed. The LWIR camera was installed on a tripod to synthesize the uniform blurry image by suppressing the roll movement. Table 1 shows the camera and gyroscope sensor parameters.

As depicted in Figure 5, in order to synthesize a blurry thermal image without the stepping effect, adjacent images should be shifted by at most one pixel. Therefore, the maximum rotation angle of a camera between two adjacent images should be limited to the angle of IFOV. Since the IFOV of a FLIR camera is 0.0391°, and the frame rate is 50 Hz, the above condition can be satisfied if the camera rotation speed should be less than 1.955°/s. Since a gyroscope measures the angular velocity of a camera, the camera rotation speed is able to keep less than 1.955°/s during image acquisition. As shown in Table 2, the total number of images in each subset of the SBI dataset is between 1400 and 2000. The gyroscope data has been stored while synchronized with sharp images. Since the gyroscope frame rate is 1000 Hz, the camera rotation motion between adjacent images has been paired with 20 consecutive gyroscope data.

**Figure 10:**
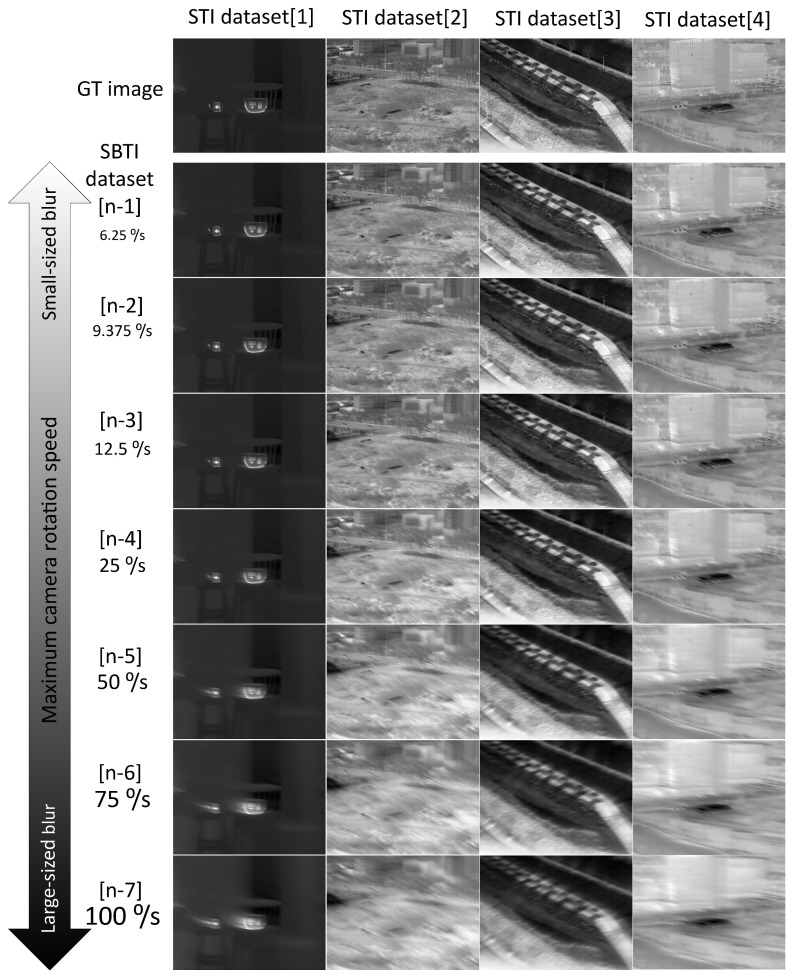
Overview of STI and SBTI datasets.

The SBTI dataset is generated through Equation (10) based on the STI dataset. In Equation (10), the blur size is determined by α which consists of τ and *h*. Here, τ is thermal time constant, and *h* is interval time between two consecutive images (where *h* is 20 ms, having camera frame rate as 50 Hz). We adjust the blur size by changing the value of *h*. The real interval time of two sharp images is 20 ms, but we can control the blur size by replacing this interval time with a specific value. For example, assuming *h* is 1/1280, the frame rate between two sharp images becomes 1280 Hz. In other words, the time consumed to collect 1280 images is no longer 25.6 s but 1 s. The camera rotation speed also is converted from 1.955°/s to 50°/s. This range is about 25.6 times higher than a real camera rotation speed. Using this time compression method, we can generate blurry images corresponding to any camera rotation speed. Finally, the blurry images are sampled every 20 frames and converted to 8-bit images for comparison. Figure 10 and Table 3 show the configurations of STI and SBTI datasets. In the SBTI dataset, there are seven different blur sizes, and the maximum camera rotation speed intuitively expresses the blur size.

### 4.2. Construction of Real Blurry Thermal Image Dataset

We collected an additional dataset containing real motion blur for evaluating our method in a real-world environment. The process for acquiring real blurry images is as same as the one for collecting sharp images as presented in Section 4, except that there is no limitation in camera rotation speed for the real effect of a blur. Another difference is that, since we use only one camera, we cannot acquire sharp images at the same time when collecting real blurry images. Specifically, the camera rotation speed varies from 30°/s to 100°/s. In addition, since infrared images are greatly affected by environmental temperature change, we collected daytime and nighttime images, respectively.

### 4.3. Our Deblurring Procedure

We evaluate the accuracy of our proposed blur kernel rendering result through the deblurring procedure. Therefore, we selected the deconvolution algorithm [65] which can be combined with blur kernel rendering result to construct a non-blind deblurring method. Actually, we used the public code version of [66] implementing [65]. In our experiment, we set parameters as follows: λ = 0.001∼0.003, α = 1.

### 4.4. Evaluation Environment

Blur kernel rendering and non-blind deblurring are implemented in MATLAB. NVIDIA GeForce GTX 1080 Ti GPU with 11 GB memory and Intel core i7-1065 G7@1.3G HZ with 16 GB memory have been adopted.

## 5. Experimental Results

Our experimental results are compared to the state-of-the-art deblurring methods, including the single image deblurring methods [33,35,36] and the deep learning-based video deblurring method [34]. We conducted both qualitative and quantitative comparisons on our SBTI dataset. Additionally, we used the real blurry thermal images to qualitatively evaluate the deblurring performance in actual situations.

### 5.1. Performance Evaluation on SBTI Dataset

The peak signal-to-noise ratio (PSNR) and structural similarity (SSIM) [67] index were leveraged as the metrics of quantitative evaluation. The experimental results are summarized in Table 4, Table 5, Table 6 and Table 7 as average values. Relatively higher PSNR and SSIM have been observed from [1-1] to [1-7] compared to the others in the SBTI dataset. As can be observed in the Table 4, Table 5, Table 6 and Table 7, PSNR and SSIM tend to gradually decrease when the blur size increases. In most cases, our proposed method produces relatively higher PSNR and SSIM values compared to the state-of-the-art methods.

**Figure 11:**
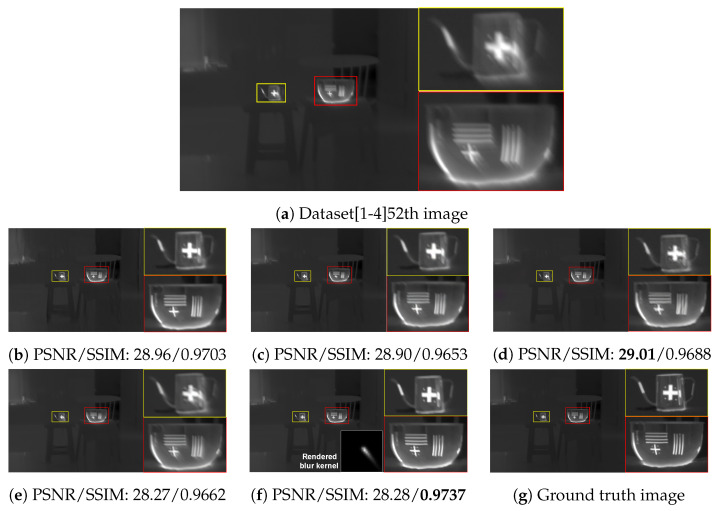
Qualitative comparison of deblurring results on the SBTI dataset [1-4]54th. (**a**) Synthetic blurry thermal image, (**b**) SRN [33], (**c**) SIUN [36], (**d**) DeblurGAN.v2 [35], (**e**) CDVD [34], (**f**) Ours, (**g**) GT.

**Figure 12:**
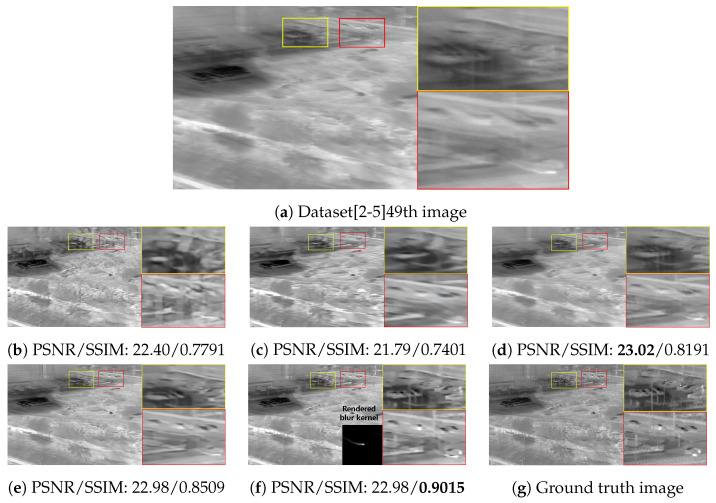
Qualitative comparison of deblurring results on the SBTI dataset [2-5]49th. (**a**) Synthetic blurry thermal image, (**b**) SRN [33], (**c**) SIUN [36], (**d**) DeblurGAN.v2 [35], (**e**) CDVD [34], (**f**) Ours, (**g**) GT.

**Figure 13:**
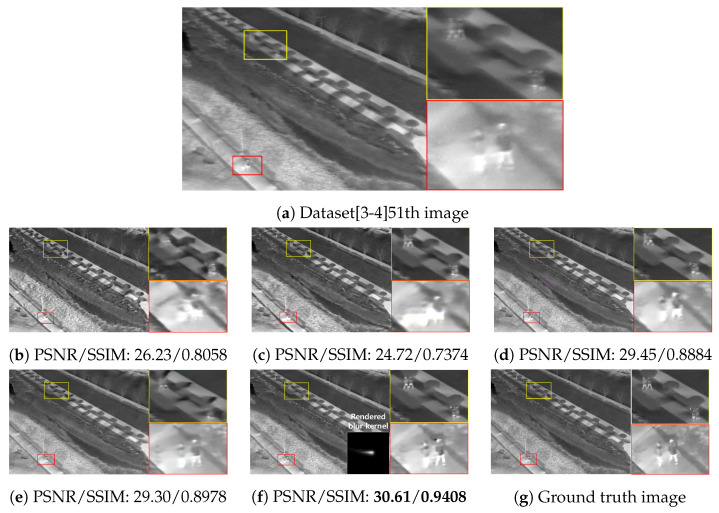
Qualitative comparison of deblurring results on the SBTI dataset [3-4]51th. (**a**) Synthetic blurry thermal images, (**b**) SRN [33] (**c**) SIUN [36], (**d**) DeblurGAN.v2 [35], (**e**) CDVD [34], (**f**) Ours, (**g**) GT.

**Figure 14:**
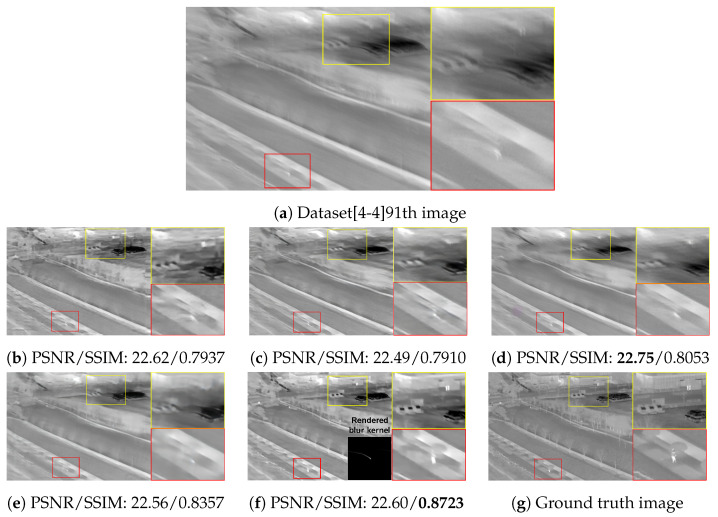
Qualitative comparison of deblurring results on the SBTI dataset [4-4]91th. (**a**) Synthetic blurry thermal image, (**b**) SRN [33], (**c**) SIUN [36], (**d**) DeblurGAN.v2 [35], (**e**) CDVD [34], (**f**) Ours, (**g**) GT.

The qualitative comparing results are shown in Figure 11, Figure 12, Figure 13 and Figure 14. Figure 11 shows the deblurring results on the 54th frame of the SBTI dataset [1-4]. The main subjects of the SBTI dataset [1-4] consist of a cross pattern and a 4-bar pattern. Unlike the other methods, which partially removed the blur effect, our proposed method dramatically recover the blur effect. The shape of the small spot at the edge of the cross-pattern reveals the signal attenuation characteristics of the blurry thermal image. This signal attenuation effect makes the small subject disappear in the blurry image. As shown in other algorithm results, it is not easy to restore the blurry image with an extreme loss of signal. In this case, the size of the blur kernel rendered by our proposed method is 20 by 20. Figure 12 shows the deblurring results on the 49th frame of the SBTI dataset [2-5], and the main subject is a group of vehicles. In this blurry image, it is difficult to recognize either the number of vehicles or their shapes. The result of SRN shows that it is almost impossible to recognize a vehicle in the deblurred image. Further, the other methods still fail to restore the shapes of vehicles due to the signal attenuation effect. In this dataset, the signal attenuation effect makes the subject and the background indistinguishable. In contrast, our result shows high restoration performance enough to recognize the number of vehicles and distinguish their external shapes. In this case, the size of the blur kernel rendered by our proposed method is 54 by 54. Figure 13 shows the deblurring results on the 51th frame of the SBTI dataset [3-4]. The main subject is people. Our method most clearly restores the shape of human arms and legs than other competing methods. Further, SRN and CDVD methods show distorted restoration results regarding the tree’s shape in the promenade center. In the case, the size of the blur kernel rendered by our proposed method is 24 by 24. Figure 14 shows the deblurring results on the 91th frame of the SBTI dataset. It is very difficult to recognize the number of subjects or their shapes without referring to the ground truth image. Our proposed method successfully restores the blurry image so that the details are sufficiently revealed, such as the number of people and the shapes of vehicles. Most people and vehicles’ edges disappeared in this blurry image due to the signal attenuation effect. It is challenging to predict the blur kernel in an image where the subject and the background cannot be distinguished. It is also difficult to show good restoration results without learnable knowledge, even using a deep learning-based approach. In the case, the size of the blur kernel rendered by our proposed method is 107 by 107.

### 5.2. Performance Evaluation on Real Blurry Thermal Images

**Figure 15:**
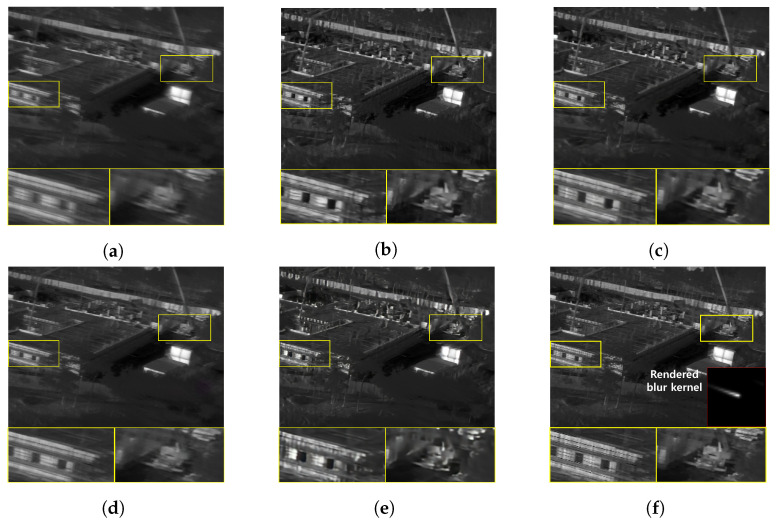
Qualitative comparison of motion deblurring results on the real blurry thermal image. (**a**) Real blurry thermal image acquired with a camera rotating at 31°/s, (**b**) SRN [33], (**c**) SIUN [36], (**d**) DeblurGAN.v2 [35], (**e**) CDVD [34], (**f**) Ours.

**Figure 16:**
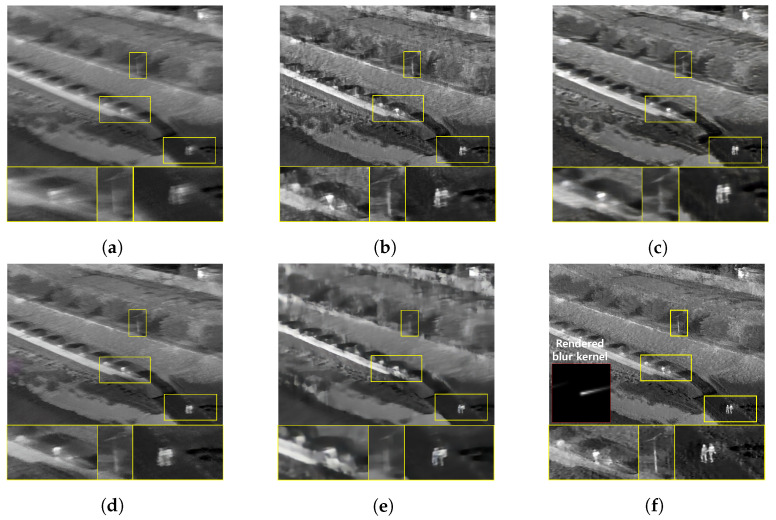
Qualitative comparison of motion deblurring results on the real blurry thermal image. (**a**) Real blurry thermal image acquired with a camera rotating at 39°/s, (**b**) SRN [33], (**c**) SIUN [36], (**d**) DeblurGAN.v2 [35], (**e**) CDVD [34], (**f**) Ours.

**Figure 17:**
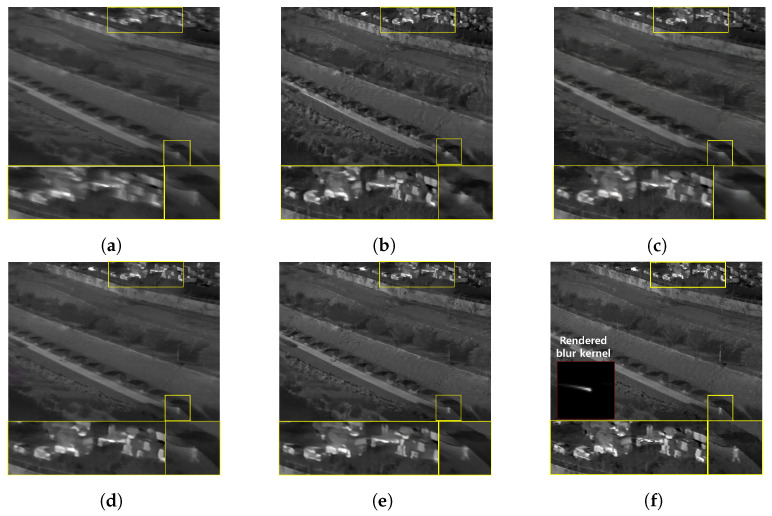
Qualitative comparison of motion deblurring results on the real blurry thermal image. (**a**) Real blurry thermal image acquired with a camera rotating at 43°/s, (**b**) SRN [33], (**c**) SIUN [36], (**d**) DeblurGAN.v2 [35], (**e**) CDVD [34], (**f**) Ours.

**Figure 18:**
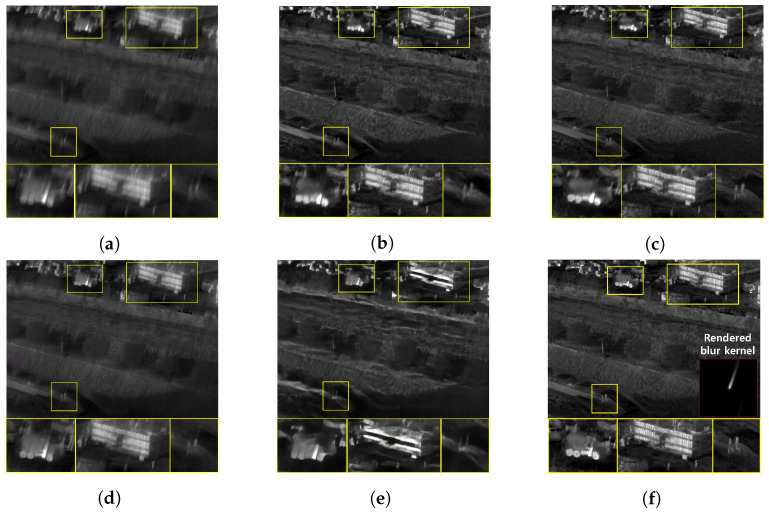
Qualitative comparison of motion deblurring results on the real blurry thermal image. (**a**) Real blurry thermal image acquired with a camera rotating at 44°/s, (**b**) SRN [33], (**c**) SIUN [36], (**d**) DeblurGAN.v2 [35], (**e**) CDVD [34], (**f**) Ours.

**Figure 19:**
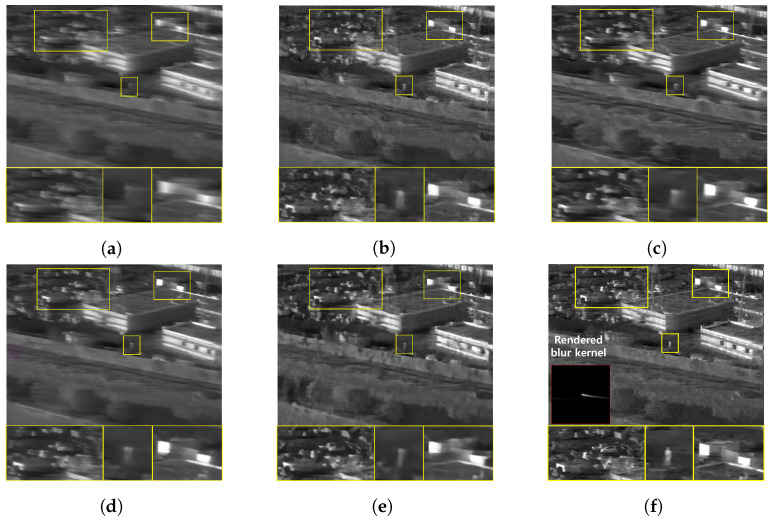
Qualitative comparison of motion deblurring results on the real blurry thermal image. (**a**) Real blurry thermal image acquired with a camera rotating at 84°/s, (**b**) SRN [33], (**c**) SIUN [36], (**d**) DeblurGAN.v2 [35], (**e**) CDVD [34], (**f**) Ours.

**Figure 20:**
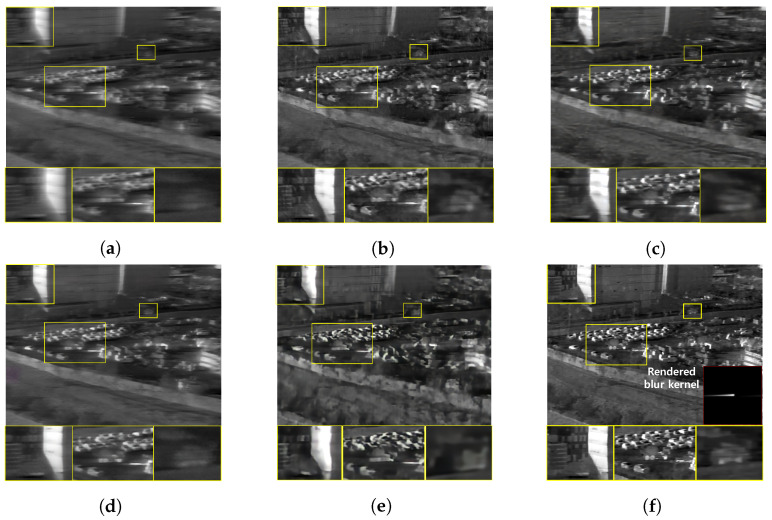
Qualitative comparison of motion deblurring results on the real blurry thermal image. (**a**) Real blurry thermal image acquired with a camera rotating at 85°/s, (**b**) SRN [33], (**c**) SIUN [36], (**d**) DeblurGAN.v2 [35], (**e**) CDVD [34], (**f**) Ours.

**Figure 21:**
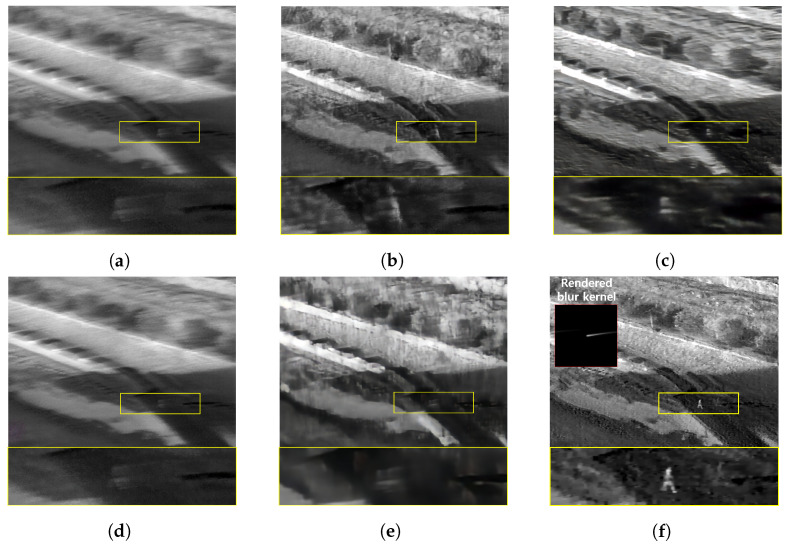
Qualitative comparison of motion deblurring results on the real blurry thermal image. (**a**) Real blurry thermal image acquired with a camera rotating at 100°/s, (**b**) SRN [33], (**c**) SIUN [36], (**d**) DeblurGAN.v2 [35], (**e**) CDVD [34], (**f**) Ours.

**Figure 22:**
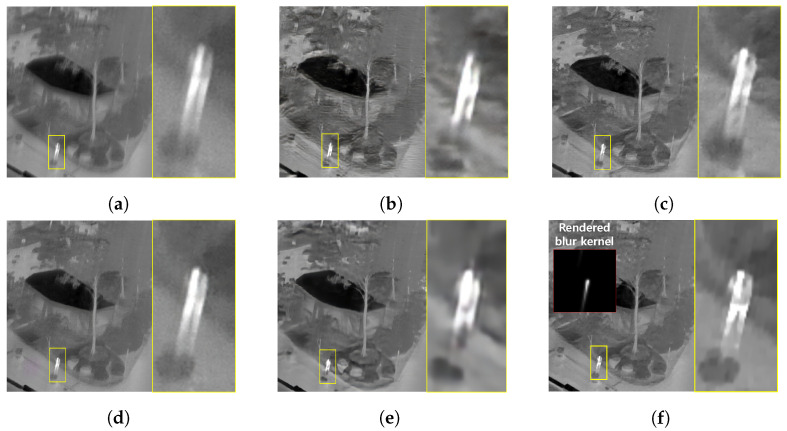
Qualitative comparison of motion deblurring results on the real blurry thermal image. (**a**) Real blurry thermal image acquired with a camera rotating at 40°/s, (**b**) SRN [33], (**c**) SIUN [36], (**d**) DeblurGAN.v2 [35], (**e**) CDVD [34], (**f**) Ours.

Furthermore, we conduct a qualitative comparison between our proposed method and other methods on real blurry images. Since the real blurry images cannot have the supplementary sharp images as ground truth, only qualitative comparisons are performed. Figure 15 and Figure 16 show the blurry thermal images of building, construction equipment and people, collected when the camera rotation speed has been about 30°/s. Even though the blur effect is low in these images, the competing algorithm results show a residual blur effect in their restoration images. In contrast, our proposed method successfully recovers blurry images, so the shape of the subject is distinguished well. Figure 17 and Figure 18 show the blurry thermal images of vehicles, buildings, and people, collected while the camera rotation speed has been about 40°/s. Because of the effect of a motion blur, we can barely know the shape of the subject in the real blurry images. As can be seen in Figure 17c and Figure 18e, the shape of a person still has the blur effect in the restoration image. On the other hand, our proposed method shows the restoration result that has the fully recognizable shape of the person’s arms and legs and contains the details of the vehicle’s wheels. Figure 19 and Figure 20 depict the results of images acquired when the camera rotation speed has been about 80°/s. Because of the large level of blur effect, it is impossible to recognize the shape or number of any subject. Although the competing methods reduced the blur effect, their restoration images are not enough to recognize the details of a subject. On the other hand, our proposed method recovers the details of subjects better than the competing methods. In Figure 21, the blurry image was obtained while the camera rotation speed has been about 100°/s. The blur effect had been so huge that the contour or presence of a subject is barely recognizable. However, our method remarkably restores the shape of a person, and all competing methods failed. Figure 22 is the image data collected at night, when the camera rotation speed has been 40°/s. Similar to the above results, our method restores the shape of a person, while the competing methods do not.

Extensive experimental results show that our proposed method outperforms other methods. The reason is that our approach is able to estimate more accurate blur kernels using a physical model and inertial sensor. There are two explanations regarding how our method can render the exact blur kernel. Firstly, our method leverages the physical mechanism of a thermal detector for accurate blur kernel rendering. As shown in Figure 2, the pixel structure of a thermal detector loses its stored thermal energy over time which appears as the effect of attenuation of an image signal. This attenuation effect causes motion blur similar to a comet tail shape. As shown in Figure 14 and Figure 17, Figure 18, Figure 19, Figure 20 and Figure 21, when a small-sized subject has its temperature similar to the background, the subject is barely distinguished from the background due to its attenuation effect of motion blur. It is extremely challenging to obtain a blur kernel from an intensely blurred image where the subject has almost disappeared. Further, even with a deep learning-based method, high performance is hardly achieved without learnable information. In contrast, our method shows high deblurring performance even for vanishing subjects with a large amount of motion blur. For this reason, our proposed method, which is designed considering the characteristics of the thermal detector, is able to show high feasibility compared to other methods in the thermal image domain. Secondly, accurate blur kernel rendering is possible since our proposed method is free from the synchronization problem between the gyroscope data length and the image sensor exposure time. In general, to combine photon detector and gyroscope data, the synchronization problem between photon detector exposure time and gyroscope sensor data length must be resolved. A photon detector adjusts the exposure time in real-time according to the amount of ambient light in a scene. The exposure time range is generally set from a few microseconds to several seconds. Due to the dynamic change in exposure time, the length of gyroscope data also needs to be changed simultaneously. In contrast, in a thermal detector, the concept corresponding to the exposure time of the photon detector is the thermal time constant. Since the thermal time constant is a fixed value determined when a thermal detector is fabricated, the length of gyroscope data used for blur kernel rendering is not changed. Therefore, a thermal detector combined with a gyroscope is more feasible to render the accurate blur kernel.

## 6. Conclusions

In this paper, we observed that a thermal detector and a photon detector have different inherent characteristics, which accordingly cause different motion blur effects. Based on this observation, we have analyzed the physical and theoretical differences between a thermal detector and a photon detector in order to precisely model a motion blur effect in the thermal image. We suggest a novel motion blur model for thermal images by interpreting the physical mechanism of a thermal detector. The proposed motion blur model is leveraged to enable blur kernel rendering to accurate using gyroscope sensor information. We constructed the first blurry thermal image dataset that contains both synthetic blurred images and sharp thermal images in the thermal image domain. Finally, extensive qualitative and quantitative experiments were conducted to show that our proposed method outperforms the state-of-the-art methods.

## Figures and Tables

**Table 1:** The Parameters of Camera-Gyroscope integrated system.

Camera Parameters		Gyroscope Parameters	
Resolution (pixel)	640 × 480	Resolution (°/s)	0.0076
Frame rate (Hz)	50	Frame rate (Hz)	1000
FOV/IFOV (°)	25 × 19/0.0391	Range (°/s)	±200
Thermal time constant (ms)	8	Bias drift (°/s)	0.12
Focal length (mm)/f/♯	24.6/1.0	Total RMS noise (°/s)	0.05

**Table 2:** Configuration of STI Dataset.

STI Dataset	Subject	# of Images	# of Gyro.	Collection Environment	Bit Depth
[1]	Test pattern	1400	28000	Indoor	16 bits
[2]	Vehicle, Road	1600	32000	Outdoor	16 bits
[3]	Person, Road	2000	40000	Outdoor	16 bits
[4]	Person, Vehicle	2000	40000	Outdoor	16 bits

**Table 3:** Configuration of SBTI dataset.

STI Dataset	SBTI Dataset						
	Maximum Camera Rotation Speed (°/s)						
	6.25	9.375	12.5	25	50	75	100
[1]	[1-1]	[1-2]	[1-3]	[1-4]	[1-5]	[1-6]	[1-7]
[2]	[2-1]	[2-2]	[2-3]	[2-4]	[2-5]	[2-6]	[2-7]
[3]	[3-1]	[3-2]	[3-3]	[3-4]	[3-5]	[3-6]	[3-7]
[4]	[4-1]	[4-2]	[4-3]	[4-4]	[4-5]	[4-6]	[4-7]

**Table 4:** Comparison of quantitative deblurring performance on the SBTI dataset [1-1]–[1-7].

SBTI Dataset	SRN [33]		SIUN [36]		DeblurGAN.v2 [35]		CDVD [34]		Ours	
	PSNR	SSIM	PSNR	SSIM	PSNR	SSIM	PSNR	SSIM	PSNR	SSIM
[1-1]	40.33	0.9881	41.03	0.9914	41.30	0.9910	39.62	0.9905	**41.57**	**0.9926**
[1-2]	37.96	0.9849	38.45	0.9889	38.37	0.9872	37.09	0.9874	**38.79**	**0.9906**
[1-3]	35.94	0.9815	36.35	0.9858	36.13	0.9835	35.05	0.9840	**36.42**	**0.9880**
[1-4]	30.97	0.9675	**31.11**	0.9714	30.91	0.9695	30.36	0.9699	31.06	**0.9756**
[1-5]	26.69	0.9419	**26.74**	0.9476	26.64	0.9456	26.32	0.9453	26.65	**0.9526**
[1-6]	24.59	0.9221	**24.67**	0.9298	24.57	0.9273	24.34	0.9271	24.52	**0.9337**
[1-7]	23.21	0.9049	**23.33**	0.9141	23.22	0.9118	23.07	0.9130	23.11	**0.9165**
Average	31.38	0.9558	31.67	0.9613	31.59	0.9594	30.84	0.9596	**31.73**	**0.9642**

**Table 5:** Comparison of quantitative deblurring performance on the SBTI dataset [2-1]–[2-7].

SBTI Dataset	SRN [33]		SIUN [36]		DeblurGAN.v2 [35]		CDVD [34]		Ours	
	PSNR	SSIM	PSNR	SSIM	PSNR	SSIM	PSNR	SSIM	PSNR	SSIM
[2-1]	28.66	0.8573	29.74	0.9026	32.25	0.9458	28.12	0.8358	**32.98**	**0.9600**
[2-2]	27.06	0.8247	27.97	0.8719	30.06	0.9221	26.54	0.8076	**30.93**	**0.9504**
[2-3]	26.02	0.8048	26.72	0.8455	28.69	0.9014	25.57	0.7891	**29.55**	**0.9396**
[2-4]	23.82	0.7603	24.32	0.7805	25.81	0.8405	24.04	0.7679	**26.38**	**0.9034**
[2-5]	21.78	0.7128	22.54	0.7421	23.36	0.7738	22.74	0.7674	**23.49**	**0.8492**
[2-6]	20.29	0.6743	21.01	0.7063	21.74	0.7262	21.53	0.7450	**21.86**	**0.8104**
[2-7]	19.11	0.6487	19.66	0.6776	20.28	0.6902	20.47	0.7204	**20.61**	**0.7757**
Average	23.82	0.7547	24.56	0.7895	26.03	0.8286	24.14	0.7762	**26.54**	**0.8841**

**Table 6:** Comparison of quantitative deblurring performance on the SBTI dataset [3-1]–[3-7].

SBTI Dataset	SRN [33]		SIUN [36]		DeblurGAN.v2 [35]		CDVD [34]		Ours	
	PSNR	SSIM	PSNR	SSIM	PSNR	SSIM	PSNR	SSIM	PSNR	SSIM
[3-1]	29.20	0.8606	29.64	0.8862	35.69	**0.9603**	34.034	0.9240	**36.556**	0.9600
[3-2]	27.93	0.8305	28.66	0.8597	33.79	0.9368	32.43	0.9081	**35.02**	**0.9525**
[3-3]	27.05	0.8053	27.92	0.8394	32.66	0.9201	31.45	0.8965	**33.95**	**0.9452**
[3-4]	25.34	0.7556	26.25	0.7961	30.10	0.8772	29.21	0.8657	**31.10**	**0.9177**
[3-5]	24.29	0.7348	24.90	0.7656	27.27	0.8237	26.72	0.8263	**28.00**	**0.8786**
[3-6]	23.38	0.7196	23.90	0.7435	25.52	0.7882	25.14	0.7982	**25.93**	**0.8427**
[3-7]	22.48	0.7034	22.94	0.7215	24.21	0.7605	23.82	0.7726	**24.53**	**0.8128**
Average	25.67	0.7728	26.32	0.8017	29.89	0.8667	28.97	0.8559	**30.73**	**0.9013**

**Table 7:** Comparison of quantitative deblurring performance on the SBTI dataset [4-1]–[4-7].

SBTI Dataset	SRN [33]		SIUN [36]		DeblurGAN.v2 [35]		CDVD [34]		Ours	
	PSNR	SSIM	PSNR	SSIM	PSNR	SSIM	PSNR	SSIM	PSNR	SSIM
[4-1]	30.37	0.8925	31.42	0.9271	33.63	0.9552	32.19	0.9258	**34.05**	**0.9640**
[4-2]	29.02	0.8742	29.78	0.9066	31.78	0.9373	30.77	0.9177	**32.34**	**0.9589**
[4-3]	28.14	0.8620	28.71	0.8900	30.67	0.9262	29.86	0.9110	**31.22**	**0.9532**
[4-4]	25.98	0.8294	26.40	0.8531	27.87	0.8923	27.44	0.8937	**28.20**	**0.9312**
[4-5]	23.88	0.7947	24.22	0.8137	**25.19**	0.8506	24.81	0.8636	25.02	**0.8956**
[4-6]	22.53	0.7731	22.82	0.7869	**23.53**	0.8216	23.22	0.8390	23.41	**0.8704**
[4-7]	21.52	0.7567	21.74	0.7662	**22.33**	0.8022	22.06	0.8175	22.30	**0.8460**
Average	25.92	0.8261	26.44	0.8491	27.86	0.8836	27.19	0.8812	**28.08**	**0.9170**

## Data Availability

Anyone who wants to use the dataset presented in this paper can receive the dataset by filling out a simple request form at the following link. Link: https://forms.gle/ZRK1R1imETkzCWkh8 (accessed on 20 January 2022).
